# Dr. William L. Allen

**Published:** 1915-03

**Authors:** 


**Dr. William L. Allen,** Buffalo, 1875; died at his home in North Tonawanda, February 17, 1915. He was born in Romulus, Seneca County, N. Y., in February 1848; was educated at the Albany State Normal School and taught for several years in Seneca Co., before studying medicine. He had served as coroner and as health officer for the town of Wheatfield. He was ill only two days.

